# Analysis of virulence profiles in clinical isolates of *Klebsiella pneumoniae* from renal abscesses: clinical significance of hypervirulent isolates

**DOI:** 10.3389/fcimb.2024.1367111

**Published:** 2024-03-28

**Authors:** Jaehyeon Lee, Jeong-Hwan Hwang, Ji Hyun Yeom, Sik Lee, Joo-Hee Hwang

**Affiliations:** ^1^ Department of Laboratory Medicine, Jeonbuk National University Medical School and Hospital, Jeonju, Republic of Korea; ^2^ Research Institute of Clinical Medicine of Jeonbuk National University—Biomedical Research Institute of Jeonbuk National University Hospital, Jeonju, Republic of Korea; ^3^ Department of Internal Medicine, Jeonbuk National University Medical School and Hospital, Jeonju, Republic of Korea

**Keywords:** *Klebsiella pneumoniae*, renal abscess, virulence, hypervirulence, antibiotic resistance

## Abstract

**Introduction:**

*Klebsiella pneumoniae* can cause a wide range of infections. Hypervirulent *K. pneumoniae* (hvKp), particularly associated with the K1 and K2 capsular types, is an increasingly significant microorganism with the potential to cause invasive infections, including renal abscesses. Despite the rising prevalence of hvKp infections, information on renal abscesses caused by *K. pneumoniae* is limited, and the clinical significance of hvKp associated with specific virulence genes remains elusive.

**Methods:**

This study performed at a 1200-bed tertiary hospital sought to identify the clinical and microbiological characteristics of renal abscesses caused by *K. pneumoniae*, focusing on various virulence genes, including capsular serotypes and multilocus sequence typing (MLST).

**Results:**

Over an 8-year period, 64 patients with suspected renal abscesses were reviewed. Ten patients diagnosed with *K. pneumoniae*-related renal abscesses were ultimately enrolled in the study. Among the isolates from the 10 patients, capsular serotype K2 was predominant (40.0%), followed by K1 (30.0%). The most common sequence type by MLST was 23 (40.0%). In particular, six patients (60.0%) harbored specific genes indicative of hvKp: *iucA*, *peg-344*, *rmpA*, and *rmpA2*.

**Conclusions:**

Our findings highlight the importance of hvKp as a pathogen in renal abscesses. Although the nature of hvKp is relatively unknown, it is widely recognized as a highly virulent pathogen that can infect relatively healthy individuals of various ages and simultaneously cause infections at multiple anatomical sites. Therefore, when treating patients with *K. pneumoniae*-related renal abscesses, caution is necessary when considering the characteristics of hvKp, such as potential bacteremia, multi-organ abscess formation, and metastatic spread.

## Introduction

A renal abscess is a rare condition that occurs within the renal parenchyma (Coelho et al., 2007). Infections leading to renal abscesses can arise from ascending infections originating in the bladder or through hematogenous spread from a primary infection site (Coelho et al., 2007; Gardiner et al., 2011). The clinical diagnosis of renal abscesses is challenging because there are no distinctive symptoms or signs specific to the condition. However, advanced diagnostic modalities, including ultrasonography, computed tomography, and magnetic resonance imaging, have enabled the prompt and precise diagnosis of renal abscesses, despite the lack of pathognomonic clinical features (Fowler and Perkins, 1994; Coelho et al., 2007).

The emergence and widespread use of antibiotics have changed the etiology of renal abscesses from primarily *Staphylococcus aureus* responsible for hematogenous spread, to predominantly Gram-negative organisms (92.3% of cases), with *Escherichia coli* (69.2%) and *Klebsiella pneumoniae* (23.1%) being the most common causative microorganisms (Lee et al., 2008).

*K. pneumoniae* is an increasingly significant microorganism with the potential to cause significant organ damage. Currently, there are two types of *K. pneumoniae* in circulation: “classic” *K. pneumoniae* (cKp) and hypervirulent *K. pneumoniae* (hvKp). Since the first description of a *K. pneumoniae* infection in 1882, cKp has been recognized as the main causative pathogen (Shon et al., 2013). However, the landscape changed with the first documented case of septic endophthalmitis associated with a pyogenic liver abscess caused by *K. pneumoniae* reported in Taiwan in 1986. Since then, hvKp has emerged as a global pathogen (Liu et al., 1986). cKp has been associated with infections involving the genitourinary tract, abdomen, lungs, endovascular system, surgical sites, and soft tissues, and has been identified as a cause of subsequent bacteremia in hospitals and long-term care facilities (Paczosa and Mecsas, 2016). Metastatic infections caused by enteric gram-negative organisms are rare without predisposing factors such as neutropenia or malignancy (Paczosa and Mecsas, 2016). cKp is an opportunistic pathogen that causes infections in immunocompromised patients, particularly in healthcare settings (Paczosa and Mecsas, 2016). Although the characteristics of hvKp and its differences from cKp remain relatively unknown, it is widely recognized that hvKp is a highly virulent pathogen that can infect individuals of varying ages who are relatively healthy, and it can cause simultaneous infections at multiple anatomical sites (Paczosa and Mecsas, 2016). There are at least 78 capsular serotypes, with K1, K2, K5, K16, K20, K54, K57, and KN1 being the most common hypervirulent serotypes (Shon et al., 2013). Recent reports have shown that serotypes K1 and K2 are primarily associated with hvKp (Paczosa and Mecsas, 2016). Although the exact definition of hvKp remains unclear, Russo et al. demonstrated that *iroB*, *iucA*, *peg-344*, *rmpA*, and *rmpA2* serve as highly discriminatory virulence factors that confer hypervirulent phenotype (Liu and Guo, 2018). To date, *iuc*, *rmpA*, and *rmpA2* are considered the most comprehensively understood molecular markers of hvKp (Russo and Marr, 2019). Therefore, based on these experimental results, we defined the isolates containing *iucA*, *rmpA*, and *rmpA2* as hvKp.

Despite the increasing prevalence of hvKp infections, particularly in Taiwan since the mid-1980s, there is limited understanding of the association between renal abscesses and *K. pneumoniae* (Chang et al., 2000; Shon et al., 2013). Additionally, the clinical significance of hvKp-related K1 or K2 capsular serotypes or virulence genes in renal abscesses remains unclear. This study aimed to thoroughly investigate the clinical and microbiological aspects of renal abscesses caused by *K. pneumoniae* based on the hypothesis that hvKp strains are likely associated with renal abscesses, particularly in relation to various virulence genes.

## Materials and methods

### Patients and data collection

A single-center retrospective study was conducted at a 1,200-bed tertiary hospital between January 2014 and December 2021. Patients 18 years of age and older who were diagnosed as a renal abscess having *K. pneumoniae* isolated from urine, pus, or blood cultures were eligible. Patients with perirenal abscesses were excluded. The diagnosis of renal abscess was confirmed by abdominopelvic computed tomography. Demographic and clinical information were collected retrospectively from electronic medical records, including age, comorbidities, clinical symptoms and sings, clinical outcomes, treatment, and laboratory data.

### Bacterial isolation, identification, and string test

All laboratory tests were performed in routine clinical practice. For cultures, all samples were inoculated to blood agar plates (POAMEDIA Blood Agar Plates, Shin Yang Chemical Co., Seoul, Republic of Korea) and MacConkey agar plates (POAMEDIA MacConkey Agar Plates, Shin Yang Chemical Co., Seoul, Republic of Korea) in a 5% CO2 incubator for 16 to 24 hours at 35°C. The BacT/Alert 3D system (bioMérieux, Durham, NC, USA) was used for blood cultures. Microorganisms were identified using the Vitek MS system (bioMérieux, Hazelwood, MI, USA). In addition, a string test of all isolates was performed for the hypermucoviscous phenotype. All isolates were incubated on blood agar plates (POAMEDIA Blood Agar Plates) for 16-18 hours. The string test is considered positive when the string is stretched more than 5 mm from a blood agar plate using an inoculation loop.

### Antimicrobial susceptibility test

The antimicrobial susceptibility tests were determined using Vitek 2 AST 211 cards (bioMérieux, Marcy-l’Étoile, France) and interpreted using the VITEK 2 identification systems (CLSI, 2018). Antimicrobials used for the test included aminoglycosides (amikacin, gentamicin), cephalosporins (cefazolin, cefotaxime, ceftazidime, cefepime), cephamycins(cefoxitin), fluoroquinolones (levofloxacin), folate pathway inhibitors (trimethoprim-sulfamethoxazole), glycylcyclines (tigecycline), monobactams (aztreonam), and penicillin + β lactamase inhibitors (ampicillin-sulbactam, piperacillin-tazobactam). Multidrug-resistant (MDR) bacteria were defined as acquired non-susceptibility to at least one agent in three or more antimicrobial categories (Magiorakos et al., 2012).

### PCR based detection of virulence and extended-spectrum beta-lactamase genes, and multilocus sequence typing

We extracted DNA from *K. pneumoniae* isolates using the boiling method and extraction buffer (Seegene, Seoul, South Korea) (Ivanov and Bachvarov, 1987). Two or three loopfuls of colonies from blood agar plates were transferred into 1 mL of distilled water contained within an Eppendorf tube. The mixture was then subjected to vortexing before being centrifuged at 13,000 rpm for 10 minutes using a microcentrifuge. Subsequently, the supernatant was carefully removed, and 100 µL of DNA extraction solution was introduced to the pellet. After a further round of vortexing, the sample was heated at 95°C for 20 minutes. Following this incubation, it was centrifuged once more at 13,000 rpm for 10 minutes in a microcentrifuge. The supernatant was used for the PCR. K1 and K2 capsular serotypes, and virulence genes were detected by multiplex PCR using the Qiagen multiplex PCR kit (Qiagen, Courtaboeuf, France). Primer sets for *magA* (*wzy*-like polymerase specific to K1 strain), K2 capsular serotype-specifying *wzi* gene, and the other virulence genes (*rmpA*, *entB*, *ybtS*, *kfu*, *iutA*, *mrkD*, and *allS*) were used as previously described (Compain et al., 2014). Other serotypes, including K5, K20, K54 and K57, and other virulence genes, including *rmpA, rmpA2, iucA*, *iroB, peg-344, peg-589, and peg-1631* were analyzed using EmeraldAmp PCR Master Mix (Takara Bio Inc., Shiga, Japan) as previously described (Liu and Guo, 2018; Russo et al., 2018).

PCR for ESBL genes was performed using two isolates that showed ESBL in antibiotic susceptibility test. Three ESBL genes, *bla*_CTX-M_, *bla*_TEM_, *bla*_SHV_, were analyzed using EmeraldAmp PCR Master Mix (Takara Bio Inc., Shiga, Japan) as previously described (Maina et al., 2012). 

All isolates were sent to Macrogen (Seoul, South Korea) for MLST, and all the sequencing and analysis were done for seven housekeeping genes (*gapA, infB, mdh, pgi, phoE, rpoB, tonB*) (Diancourt et al., 2005). Maximum parsimony tree analysis of MLST results was obtained using BioNumerics (Version 7.6.3, bioMérieux, Sint-Martens-Latem, Belgium). 

## Results

In this study, we aimed to analyze the virulence profiles and antibiotic resistance of clinical isolates from patients with renal abscesses caused by *K. pneumoniae*. During the study period, 64 patients were suspected to have a renal abscess. Among them, 10 (15.6%, 10/64) patients with renal abscesses caused by *K. pneumoniae* were identified and resulting in a final total of 10 patients included in the study. The most common pathogen causing renal abscess was *E. coli*, followed by *K. pneumoniae* and *S. aureus*, respectively.

### Characteristics of patients with renal abscess caused by *K. pneumoniae*

The median patient age was 70 years (interquartile range, 59–74 years). Fever occurred in almost all patients, and abdominal pain was present in only two patients (20.0%). A total of nine patients (90.0%) had comorbidities, including diabetes mellitus (DM) in eight patients (80.0%) and alcoholic liver cirrhosis in three patients (30.0%). All 10 patients had community-acquired infections, with *K. pneumoniae* identified in urine or pus culture. Six patients (60.0%) had bacteremia. At the time of diagnosis, none of the patients had metastases to organs other than the kidneys. All patients recovered fully, with no recurrences. Laboratory data revealed pyuria in all patients. The demographic and clinical characteristics of the 10 enrolled patients are summarized in **Table 1**.

**Table 1:** Clinical characteristics of the patients with renal abscess caused by *Klebsiella pneumoniae*.

	Case 1	Case 2	Case 3	Case 4	Case 5	Case 6	Case 7	Case 8	Case 9	Case 10
**Age**	71	58	22	75	76	70	83	69	52	63
**Sex**	Male	Male	Female	Female	Female	Male	Female	Female	Male	Female
**Underlying disease**	DM	Alcoholic LC	None	DM	DM	DM, ESRD c HD	DM, Parkinson’s disease, CVA	DM, Alcoholic LC	DM, Alcohlic LC	DM, s/p LDKT
**Type of infection**	Community acquired	Community acquired	Community acquired	Community acquired	Community acquired	Community acquired	Community acquired	Community acquired	Community acquired	Community acquired
**Tissue or Pus culture**	KP	None	None	KP	KP	None	None	None	None	KP
**Urine culture**	KP	KP	KP	KP	No growth	KP	KP	KP	KP	No growth
**Bacteremia**	No	Yes	Yes	Yes	No	Yes	No	Yes	Yes	No
**Abscess side**	Right	Left	Right	Left	Both	Left	Left	Both	Right	transplanted kidney
**Other involved sites**	None	None	None	None	None	None	None	None	Prostate, Rt iliopsoas muscle	None
**Chief complaint**	Fever	Fever	RUQ pain, Fever	Fever, dyspnea	Fever	Fever	General weakness	Fever	Fever	RLQ pain, Fever
**Antibiotics**	Ceftriaxone IV, Ciprofloxacin PO	Imipenem/cilastatin IV, Cefixime PO	Piperacillin/tazobactam IV, Ciprofloxacin PO	Ceftriaxone IV, Cefixime PO	Ceftriaxone IV, Cefixime PO	Meropenem IV	Ceftriaxone IV, Cefixime PO	Ceftriaxone IV, Cefixime PO	Ceftriaxone IV, Levofloxacin PO	Ceftriaxone IV, Cefixime PO
**Procedures or Operations**	PCD(kidney)	None	None	PCD(kidney)	PCD(kidney)	PCD(kidney)	None	None	PCD(kidney)	PCD(transplanted kidney)
**Outcome**	Recovered	Recovered	Recovered	Recovered	Recovered	Recovered	Recovered	Recovered	Recovered	Recovered
**Sequalae**	None	None	None	None	None	None	None	None	None	None
**Recurrence**	None	None	None	None	None	None	None	None	None	None
Urinalysis (normal range)										
Protein (Neg)	1+	2+	1+	3+	TR	2+	Neg	1+	1+	Neg
RBC (1-4/HPF)	1-4	>30	>30	5-9	1-4	>30	0-2	1-4	0-2	0-2
WBC (1-4/HPF)	>30	>30	5-9	>30	>30	>30	21-50	>50	21-50	>50
Laboratory										
WBC (×10^3^/mm^3^)	159900	4800	14700	5230	16930	13210	12800	15080	13300	11990
Hb (g/dL)	11.0	9.5	12.9	10.7	11.0	10.3	11.7	9.6	9.8	9.9
Platelets (×10^3^/mm^3^)	270	44	201	168	353	119	154	50	93	296
ESR (mm/hr)	95	6	26	87	64	20	53	47	110	86
CRP (mg/L)	87.59	146.84	102.34	162.39	210.51	143.41	279.37	132.02	274.08	92.44
BUN (mg/dL)	24	57	7	20	15	29	28	39	41	48
Creatinine (mg/dL)	0.81	2.11	0.46	0.85	0.45	3.03	1.47	1.11	1.27	1.84
eGFR (mL/min/1.7)	89.4	33.5	141.7	66.8	97	19.9	57.8	50.8	64.5	28.8
AST (IU/L)	26	117	61	51	10	36	17	42	67	26
ALT (IU/L)	20	30	47	38	6	16	8	30	67	35
Total bilirubin (mg/dL)	0.70	2.13	1.32	0.82	0.43	0.80	0.51	0.75	2.33	0.40

ALT, alanine aminotransferase; AST, aspartate aminotransferase; BUN, blood urea nitrogen; CRP, C-reactive protein; DM, diabetes mellitus; ESR, erythrocyte sedimentation rate; Hb, hemoglobin; HPF, high-power field; IV, intravenous; KP, *K. pneumoniae*; LC, liver cirrhosis; PCD, percutaneous catheter drainage; PO, per os; RBC, red blood cells; RLQ, right lower quadrant; RUQ, right upper quadrant; TR, trace; WBC, white blood cells.

### Phenotypic/genotypic characteristics of the *K. pneumoniae* isolates

**Figure 1:**
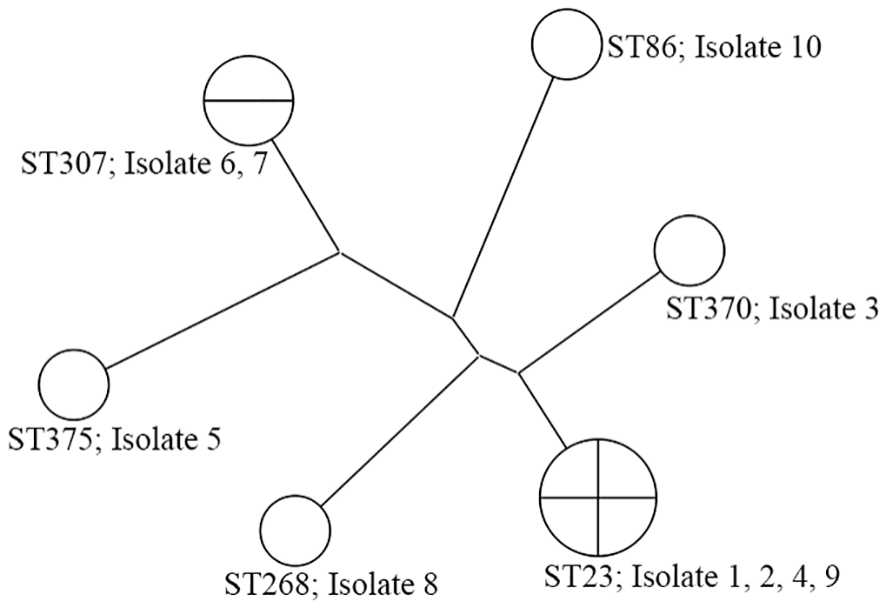
The phylogenetic analysis of 10 K*. pneumoniae* isolates from renal abscess patients. Isolates 1, 2, 4, 5, 9, and 10 are hvKp strains.

The K2 and K1 capsular serotype were detected in four (40.0%) and three isolates (30.0%), respectively. Additionally, colonies from four isolates displayed a positive string test. The MLST data of all isolates are shown in **Table 2**, and the cluster analysis according to the maximum parsimony tree results is shown in **Figure 1**. Sequence type (ST)23 was the dominant type (40.0%). Of the seven isolates with K1 or K2 capsular serotypes, ST23 was predominant and present in four (57.1%). ST23 was not found in unidentified capsular serotypes. In addition, isolates recovered from eight patients (80.0%) carried either *rmpA* or *rmpA2*, seven isolates (70.0%) carried *iucA*, and six isolates (60.0%) carried *peg-344* and *iroB* genes that identified hvKp (**Table 2**). According to our definition based on generally accepted findings, 60.0% were hvKp strains (**Table 2**).

**Table 2:** Virulence factors and serotypes of *K. pneumoniae* isolates from renal abscess patients.

Isolates Virulence factors	1	2	3	4	5	6	7	8	9	10
*rmpA*	+	+	–	+	+	–	–	–	+	+
*rmpA*2	+	+	+	+	+	–	–	+	+	+
*entB*	+	+	+	+	+	+	+	+	+	+
*ybtS*	+	+	+	+	–	+	–	+	+	–
*kfu*	+	+	+	+	–	–	–	–	+	–
*iucA*	+	+	–	+	+	–	–	+	+	+
*iutA*	+	+	–	+	–	+	+	–	+	+
*iroB*	+	+	–	+	+	–	–	–	+	+
*mrkD*	+	+	+	+	+	+	–	+	+	+
*allS*	+	+	–	+	–	–	–	+	+	+
*peg-344*	+	+	–	+	+	–	–	–	+	+
*peg-589*	+	+	–	+	+	–	–	+	+	+
*peg-1631*	+	+	–	+	+	–	–	–	–	+
String test	+	+	–	–	–	–	–	–	+	+
Serotypes	K2	K1	K1	K1	K2	Unidentified^*^	Unidentified^*^	Unidentified^*^	K2	K2
MLST	23	23	370	23	375	307	307	268	23	86
ESBL	–	–	–	–	–	+	+	–	–	–


MLST, multilocus sequence typing; ESBL, extended-spectrum beta-lactamase.

^*^Negative for K1, K2, K5, K20, K54, and K57.

### Antimicrobial susceptibility test

Eight (80.0%) *K. pneumoniae* strains were susceptible to most of the antibiotics tested. Two were identified as MDR. Among the isolates classified as hvKp, none were MDR. Among aminoglycosides, 10% of the isolates were resistant to gentamicin but not to amikacin. For cefoxitin, 30% of isolates were indeterminate. Among the cephalosporins, all tested antibiotics were resistant in 20% of the isolates. None of the carbapenems showed resistance in all isolates. Levofloxacin, trimethoprim-sulfamethoxazole, and aztreonam each showed resistance in 20% of the isolates. Tigecycline was resistant in 10% of isolates and indeterminate in 10%. Ampicillin-sulbactam was resistant in 20% of isolates and indeterminate in 10%. Two isolates were identified as ESBL-producing strains and were positive for all three ESBL gene tested; *bla*_CTX-M_, *bla*_TEM_, *bla*_SHV_. None of the isolates classified as hvKp exhibited ESBL production. The details are presented in **Table 3**.

**Table 3:** Antibiotic resistance of *K. pneumoniae* isolates from renal abscess patients.

Isolates Antimicrobials	1	2	3	4	5	6	7	8	9	10
Aminoglycosides										
Amikacin	S	S	S	S	S	S	S	S	S	S
Gentamicin	S	S	S	S	S	R	S	S	S	S
Cephamycins										
Cefoxitin	I	S	S	S	S	I	I	S	S	S
Cephalosporins										
Cefazolin	S	S	S	S	S	R	R	S	S	S
Cefotaxime	S	S	S	S	S	R	R	S	S	S
Ceftazidime	S	S	S	S	S	R	R	S	S	S
Cefepime	S	S	S	S	S	R	R	S	S	S
Carbapenems										
Ertapenem	S	S	S	S	S	S	S	S	S	S
Meropenem	S	S	S	S	S	S	S	S	S	S
Fluoroquinolones										
Levofloxacin	S	S	S	S	S	R	R	S	S	S
Folate pathway inhibitors										
TMP-SMX	S	S	S	S	S	R	R	S	S	S
Glycylcyclines										
Tigecycline	I	S	S	S	S	R	S	S	S	S
Monobactam										
Aztreonam	S	S	S	S	S	R	R	S	S	S
Penicillin + β lactamase inhibitors										
Amp-sulb	I	S	S	S	S	R	R	S	S	S
Pip-tazo	S	S	S	S	S	I	I	S	S	S

*S, susceptible; I, indeterminate; R, resistant.

**Amp-sulb, ampicillin/sulbactam; TMP-SMX, trimethoprim/sulfamethoxazole; Pip-tazo, piperacillin/tazobactam.

## Discussion

In this study, 10 cases of renal abscesses caused by *K. pneumoniae* were observed among 64 patients with renal abscesses. *K. pneumoniae* was the second most common pathogen causing renal abscesses, consistent with previous reports (Coelho et al., 2007; Lee et al., 2008). Most *K. pneumoniae*-related renal abscess cases have either K1 or K2 capsular serotypes, with K2 predominant, and 60.0% (6/10) were classified as hvKp in this study. Interestingly, in a previous report on prostatic abscess caused by *K. pneumoniae*, all isolates had the K1 capsular serotype, with 80.0% classified as hvKp (Hwang et al., 2022). This study highlights the significance of *K. pneumoniae*, particularly the hypervirulent variant, as a notable pathogen causing abscess-like complications in genitourinary infections. Although further studies are needed to generalize these findings, it is interesting to note that there is a difference in the dominant serotype between prostatic and renal abscesses caused by *K. pneumoniae*, a genitourinary infection. To the best of our knowledge, this is the first study to investigate the role of K1/K2 capsular serotypes and virulence factors in *K. pneumoniae*-related renal abscesses.

The endemicity of hvKp is currently limited to Asian countries, and the STs in which it is prevalent are ST23, ST26, ST57, ST65, ST86, ST163, and ST375. ST23 is the most prevalent among hvKp isolates, with a prevalence of 30–85% (Teo et al., 2024). We also performed MLST on all isolates. Our hvKp isolates also belong to ST23, ST86, and ST375, with ST23 being the most prevalent. In a previous study, most K1 isolates belonged to ST23, whereas K2 isolates were classified into more than 10 ST types, with ST65/ST86 predominant (Liao et al., 2014). In this study, we observed that ST23 is primarily associated with K1, but also with K2. Although this study cannot be generalized due to its small sample size, it shows both similarities and differences compared to the previous study (Liao et al., 2014). These variations may be attributed to geographic differences; however, definitive conclusions remain elusive.

Until recently, a clear definition of hvKp has been controversial. hvKp is defined by a combination of clinical and bacterial phenotypic features and is characterized by its ability to metastasize and cause invasive infections, even in otherwise healthy hosts (Shon et al., 2013). Hypervirulence is believed to be the outcome of complex interactions between multiple genetic determinants rather than stemming from a single gene (Catalán-Nájera et al., 2017; Russo and Marr, 2019). Based on previous studies, *iuc, rmpA*, and *rmpA2* have emerged as the most comprehensively understood virulence factors to date. The functions of *rmpA* and *rmpA2* may be redundant; therefore, *iuc* and either *rmpA* or *rmpA2* were expected to be closely associated with the hvKp strain (Russo and Marr, 2019). Building on previous research, our study revealed that 60.0% (6/10) of the strains identified as hvKp were responsible for renal abscesses. Despite the limited sample size, these findings suggest that hvKp is a more prevalent cause of renal abscesses than cKp.

Invasive community-acquired infections can also be caused by hvKp even in healthy adults and frequently involve multiple anatomical sites (Shon et al., 2013). In our study, all cases of renal abscesses attributed to hvKp were community-acquired infections. Among the six patients with hvKp, comorbidities were prevalent, including DM (83.3%), alcoholic liver cirrhosis (33.3%), and renal disease (16.7%), consistent with previous (Ko et al., 2002; Tsay et al., 2002). Bacteremia, multi-organ abscess formation, and metastatic spread were consistent with the well-known characteristics of hvKp (Shon et al., 2013). Our study showed that 50.0% (3/6) of patients with hvKp strains had bacteremia, with one patient showing multi-organ involvement in the prostate and right psoas muscle. Based on this and prior data, it is recommended to evaluate male patients with renal abscess and bacteremia for prostatitis or prostatic abscess (Hwang et al., 2022).

*Klebsiella* species are well known for their multidrug resistance, including the emergence of carbapenemase-producing (CP) strains and the increasing prevalence of ESBL-producing *Klebsiella*. Fortunately, most hvKp strains are susceptible to a broad spectrum of antimicrobial agents (Paczosa and Mecsas, 2016). However, recent reports have indicated an increasing trend of antimicrobial resistance among hvKp strains, including the first documented cases of CP hvKp in the United States in 2019 (Karlsson et al., 2019). Prior to this study, 12.3% of isolates in China from 2013 to 2017 were identified as CP hvKp (Liu et al., 2019). Recent GenBank data showed a 5.6% positivity rate for CP hvKp (Hu et al., 2021). Importantly, it is worth noting that none of the isolates classified as hvKp in this study were ESBL-producing or MDR. These findings provide valuable insights into the antimicrobial profiles of *K. pneumoniae* strains in the context of our study. Notably, long-term use of antimicrobial agents is a common approach for treating renal abscesses (Yen et al., 1999). The emergence of ESBL- or CP hvKp strains may limit the selection of effective antimicrobial agents for the treatment of renal abscesses and potentially lead to treatment challenges. The acquisition of resistance genes by hvKp through horizontal gene transfer involves various mechanisms, including resistance gene capture by virulence plasmid via intermolecular replicative transposition, acquisition of conjugative resistance plasmid, acquisition of resistance plasmid with unknown mechanism of conjugal transfer, and acquisition of resistance genes through other unknown mechanisms (Dong et al., 2022). These mutations confer resistance to a wide range of antibiotics (Dong et al., 2022). Multidrug resistance has increased worldwide and is considered a threat to public health (Algammal et al., 2023). These facts highlight the essential need for concurrent monitoring of hvKp and antimicrobial resistance.

We revealed the potential clinical characteristics of patients with renal abscess caused by *K. pneumoniae*, including hvKp strains. Nevertheless, this study has several limitations. As it was a single-center study with a small sample size, the results should be interpreted with caution and should not be universally applicable. Despite these constraints, our study has significant clinical relevance. Through investigations focusing on K1/K2 capsular types, MLST sequence types, and hypervirulent genes, we observed a substantial prevalence of hvKp strains in patients with renal abscesses caused by *K. pneumoniae*.

In summary, our results show that most *K. pneumoniae*-related renal abscess cases were associated with hvKp (60.0%), predominantly carrying the K2 capsular serotype and ST23. These results emphasize the significance of hvKp as a pathogen in renal abscesses, given the invasive nature of hvKp strains and their ability to invade multiple organs. Therefore, when managing patients with *K. pneumoniae*-related renal abscesses, it is important to be vigilant of hvKp characteristics, including bacteremia. Although multi-organ abscess formation and metastatic spread were identified in only one case in this study, their consideration is still essential.

## Data availability statement

The original contributions presented in the study are included in the article/supplementary material. Further inquiries can be directed to the corresponding author.

## Ethics statement

The Institutional Review Board (IRB) of Jeonbuk National University Hospital approved the protocol and waived the requirement for informed consent (IRB registration number: 2023-04-018). The studies were conducted in accordance with the local legislation and institutional requirements. The human samples used in this study were acquired from a by- product of routine care or industry. Written informed consent for participation was not required from the participants or the participants’ legal guardians/next of kin in accordance with the national legislation and institutional requirements.

## Author contributions

JL: Data curation, Writing – original draft, Writing – review & editing, Methodology, Resources. Je-HH: Data curation, Writing – original draft, Writing – review & editing, Conceptualization, Validation. JY: Validation, Writing – review & editing, Visualization. SL: Validation, Writing – review & editing, Supervision. Jo-HH: Writing – review & editing, Data curation, Formal Analysis, Funding acquisition, Writing – original draft.
